# Acute Effects of Fusion Pacing Versus Standard CRT on Myocardial Function in Heart Failure Patients with LBBB

**DOI:** 10.3390/jcm14134433

**Published:** 2025-06-22

**Authors:** Michał Kucio, Andrzej Kułach, Tomasz Skowerski, Mariusz Bałys, Mariusz Skowerski, Grzegorz Smolka

**Affiliations:** 1 Division of Cardiology, Upper Silesian Medical Center, 40-635 Katowice, Poland; 2 Department of Cardiology, School of Health Sciences in Katowice, Medical University of Silesia, 40-752 Katowice, Poland; andrzejkulach@gmail.com (A.K.);

**Keywords:** chronic resynchronization therapy, fusion pacing, biventricular pacing, atrial strains, left ventricle strains

## Abstract

**Background/Objectives:** Although cardiac resynchronization therapy (CRT) plays an established role in the management of heart failure, a significant proportion of patients do not respond despite appropriate candidate selection. The optimization of CRT pacing is one strategy to enhance response. Fusion pacing algorithms aim to synchronize intrinsic right ventricular (RV) conduction with paced left ventricular (LV) activation, resulting in a more physiological ventricular depolarization pattern. This approach may improve electrical synchrony and enhance left ventricular contraction compared to conventional simultaneous biventricular pacing. The aim of this study was to compare the acute, beat-to-beat effects of standard biventricular pacing versus fusion pacing on myocardial function, using both conventional and speckle-tracking echocardiography in heart failure patients with left bundle branch block (LBBB). **Methods**: In total, 27 heart failure patients (21 men and 6 women) with reduced ejection fraction (EF < 35%), left bundle branch block (QRS > 150 ms), and newly implanted CRT-D systems (Abbott) underwent echocardiographic assessment immediately after device implantation. Echocardiographic parameters—including left atrial strain, left ventricular strain, TAPSE, mitral and tricuspid valve function, and cardiac output—were measured at 5 min intervals under three different pacing conditions: pacing off, simultaneous biventricular pacing, and fusion pacing using Abbott’s SyncAV^®^ algorithm. **Results**: In our study, CRT led to a significant shortening of the QRS duration from 169 ± 19 ms at baseline to 131 ± 17 ms with standard biventricular pacing, and further to 118 ± 16 ms with fusion pacing (*p* < 0.05). Despite the electrical improvement, no significant changes were observed in global longitudinal strain (GLS: −9.15 vs. −9.39 vs. −9.13; *p* = NS), left ventricular stroke volume (67.5 mL vs. 68.4 mL vs. 68.5 mL; *p* = NS), or left atrial parameters including strain, area, and ejection fraction. However, fusion pacing was associated with more homogeneous segmental strain patterns, improved aortic valve closure time, and enhanced right ventricular function as reflected by tissue Doppler-derived S’. **Conclusions**: Immediate QRS narrowing observed in CRT patients—particularly with fusion pacing optimization—is associated with a more homogeneous pattern of left ventricular contractility and improvements in selected measures of mechanical synchrony. However, these acute electrical changes do not translate into immediate improvements in stroke volume, global LV strain, or left atrial function. Longer-term follow-up is needed to determine whether the electrical benefits of CRT, especially with fusion pacing, lead to meaningful hemodynamic improvements.

## 1. Introduction

Cardiac resynchronization therapy (CRT), introduced in the 1990s, is a well-established treatment for chronic heart failure of both ischemic and non-ischemic origin [1,2,3,4,5]. Its therapeutic effect is based on the simultaneous activation of the left ventricular myocardium, which improves both systolic and diastolic function and helps prevent adverse cardiac remodeling. Left ventricular stimulation is achieved by positioning a pacing lead in the lateral vein of the heart, branching from the coronary sinus, which targets the lateral wall of the left ventricle—the site of latest activation in patients with left bundle branch block (LBBB).

Current guidelines recommend CRT in patients with chronic heart failure (EF < 35%, NYHA class II–III) who have LBBB with QRS duration > 130 ms (ideally > 150 ms), or non-LBBB with QRS duration > 130 ms (ideally > 150 ms) [1]. Although echocardiographic assessment of mechanical dyssynchrony is no longer required for CRT eligibility [1,6], it may serve as a predictor of response and is often used to optimize device programming for maximal hemodynamic benefit [3,4,5,7,8].

Despite proven clinical benefits, approximately 30–40% of CRT recipients do not respond to therapy [2,7]. Contributing factors include suboptimal lead placement, myocardial scarring at the stimulation site, the absence of mechanical dyssynchrony despite QRS prolongation, comorbidities such as diabetes or renal dysfunction, and inadequate pacing percentages (<98%). This substantial non-responder rate has prompted the development of more advanced pacing algorithms.

Abbott CRT devices incorporate the SyncAV^®^ algorithm, which facilitates fusion pacing by combining intrinsic right ventricular activation with timed left ventricular pacing. This strategy results in further QRS narrowing and improved synchrony of left ventricular contraction compared to conventional simultaneous biventricular pacing. Fusion pacing is achieved through periodic measurements of native atrioventricular conduction to dynamically adjust the timing of LV pacing [9,10,11,12].

## 2. Materials and Methods

The aim of this study was to evaluate the impact of different pacing modalities on myocardial function, assessed using both echocardiography and electrocardiography. During the study, the pacing mode was sequentially modified in each patient. The analyzed parameters included QRS duration, left atrial strain, global longitudinal strain (GLS) of the left ventricle, tricuspid annular plane systolic excursion (TAPSE), mitral and tricuspid valve function, stroke volume (SV), and cardiac output (CO).

This study included 27 consecutive patients (21 men and 6 women) with heart failure with reduced ejection fraction (HFrEF), baseline left ventricular ejection fraction < 35%, left bundle branch block (QRS duration > 160 ms), and sinus rhythm. All patients were implanted with the Quadra Assura^®^ CRT-D system and a Quartet^®^ left ventricular lead (Abbott, Chicago, IL, USA) [1]. Echocardiographic assessments were performed using a Vivid E95 ultrasound system (GE Healthcare, Chicago, IL, USA). On the day after CRT-D implantation, during standard device parameter assessment, each patient underwent evaluation under three pacing conditions: pacing off, simultaneous biventricular pacing with standard atrioventricular intervals (paced AV interval: 150 ms; sensed AV interval: 120 ms), and fusion pacing using the SyncAV^®^ algorithm (minimum AV interval: 70 ms; delta: −50 ms). QRS duration was determined by measuring the interval from the pacing spike to the terminal point of the QRS complex on a standard 12-lead ECG.

**Figure 1:**
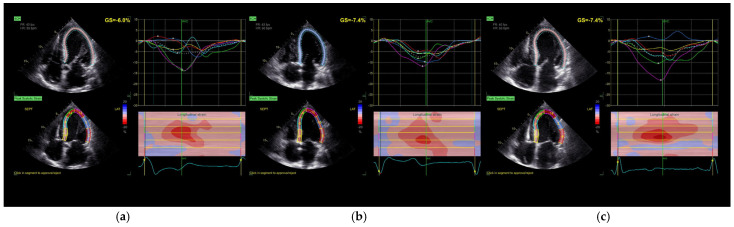
Longitudinal strain assessment in the four-chamber view: (**a**) without stimulation; (**b**) BiV stimulation; (**c**) SyncAV stimulation. GS—global longitudinal strain; AVC—aortic valve closure.

**Figure 2:**
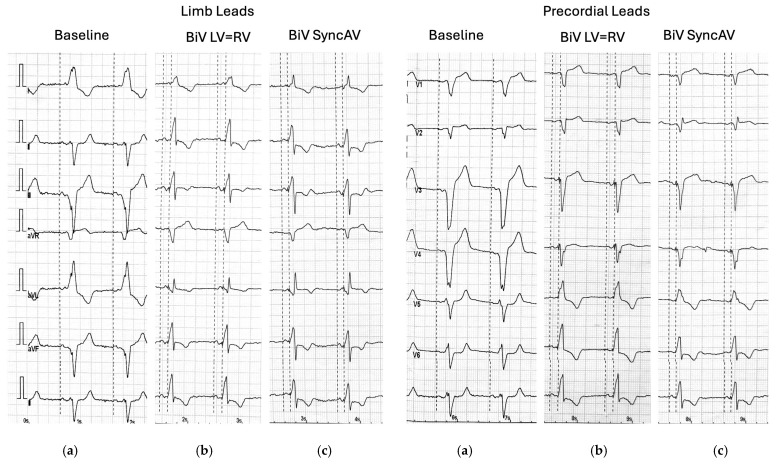
Twelve-lead ECG tracings from a representative patient with LBBB under three pacing conditions: (**a**) baseline (no pacing), (**b**) biventricular pacing (BiV LV = RV), and (**c**) fusion pacing with SyncAV^®^.

Echocardiographic measurements were taken for each pacing mode, at least 2 min after applying the setting at the same, constant heart rate. Comprehensive transthoracic echocardiography, including two-dimensional (2D), Doppler, and speckle-tracking modalities, was performed using a commercially available ultrasound system (Vivid E95, GE Healthcare) with standard phased-array transducers. Image acquisition and analysis were conducted in accordance with the guidelines and recommendations of the American Society of Echocardiography (ASE) [13,14,15,16]. Standard parasternal short-axis views at the basal, mid-ventricular, and apical levels, as well as apical four-chamber, two-chamber, and long-axis views, were acquired to fully visualize the left ventricle (LV). Pulsed-wave Doppler was used to evaluate LV inflow and outflow, and tissue Doppler imaging (TDI) was applied to assess mitral annular velocities (Figure 1 and Figure 2).

Global longitudinal strain (GLS) was assessed using two-dimensional speckle-tracking echocardiography (2D-STE) with the Automated Functional Imaging (AFI) module in EchoPAC software (version 203, GE Healthcare). Longitudinal strain was quantified by automatic endocardial border detection across the three apical views. The software generated both segmental and global strain values. Tracking quality was visually verified for all segments; manual adjustments were made when necessary to optimize contour alignment. Left atrial (LA) volumes and strain were assessed using semi-automated three-dimensional segmentation with the Auto LAQ tool. Parameters included left atrial reservoir function and ejection fraction. All echocardiographic examinations were independently performed and analyzed by two experienced echocardiographers, blinded to the pacing mode at the time of image acquisition. The reproducibility of GLS and left atrial strain measurements was qualitatively verified by two experienced echocardiographers. No formal inter- or intra-observer variability assessment was conducted.

Statistical analysis was performed with MedCalc Statistical Software version 22.026 (MedCalc Software Ltd., Ostend, Belgium). The categorical variables are reported as counts and percentages. The continuous variables are presented as the mean ± SD (standard deviation) for normal distribution and the median and IQR for non-normal distribution. Normality was tested with the Shapiro–Wilk test. In cases of normal distribution, we used ANOVA, while non-normally distributed variables were compared using Friednan ANOVA with a post hoc multiple comparison test. Qualitative parameters were compared using Pearson’s chi-square and McNemar’s tests.

The universal *p*-value level < 0.05 was considered statistically significant throughout the analyses. This investigation was carried out in accordance with the principles outlined in the Declaration of Helsinki.

## 3. Results

The baseline characteristics of the study population are presented in Table 1. The group consisted of 27 patients with reduced ejection fraction (median LVEF 30%) and a wide QRS complex (mean 172 ± 17.5 ms). The majority were male (78%) and had ischemic cardiomyopathy (56%). Nearly all patients had hypertension, and NYHA class II or III symptoms were equally represented.

The primary findings of the study are summarized in Table 2. Both simultaneous biventricular pacing and pacing with the SyncAV^®^ algorithm resulted in a statistically significant reduction in QRS duration compared to intrinsic conduction. Moreover, pacing with SyncAV^®^ produced the narrowest QRS complexes (median 120 ms), indicating superior electrical synchrony (*p* < 0.001; post hoc 1 > 2 > 3).

Despite this electrical improvement, pacing mode had no significant effect on left atrial function. Neither left atrial ejection fraction (LA EF) nor markers of left atrial stiffness (E/e’ average) differed significantly across pacing modes (*p* = NS).

Global longitudinal strain (GLS) of the left ventricle did not show statistically significant changes in response to either pacing strategy (*p* = 0.212), although a mild trend toward improvement was noted following biventricular pacing. Importantly, the time to peak longitudinal strain (Peak SL) was significantly reduced under both pacing modes compared to baseline (*p* = 0.015), reflecting improved mechanical synchrony. Additionally, SyncAV^®^ pacing was associated with a prolongation of aortic valve closure time, suggesting enhanced contractile force [17].

Diastolic function parameters showed a non-significant trend toward improvement with dual-chamber pacing, with no discernible difference between BiV and SyncAV^®^ modes.

Right ventricular function, as assessed by the S′ parameter, improved significantly during both pacing strategies. However, TAPSE values did not show significant differences. There was no significant difference between the two pacing algorithms in their impact on right ventricular performance.

## 4. Discussion

Our study assessed the acute effects of fusion pacing using the SyncAV^®^ algorithm in comparison to standard biventricular (BiV) pacing on myocardial function in CRT candidates with LBBB. While SyncAV resulted in greater QRS narrowing than BiV pacing, these changes were not accompanied by immediate improvements in key echocardiographic parameters.

### 4.1. QRS Duration and Electrical Synchrony

The SyncAV algorithm effectively shortened the QRS duration compared to BiV pacing. This observation is consistent with earlier studies that evaluated electrical optimization through fusion pacing. Martin et al., in the adaptive CRT trial, demonstrated that algorithms modulating AV delay based on intrinsic conduction may yield superior electrical synchronization compared to fixed BiV pacing [17]. A recent large-scale CRT pacing analysis by Thibault et al. (2025) further demonstrated that dynamic AV delay algorithms such as SyncAV significantly enhance electrical synchrony, especially when combined with multi-point or LV-only pacing strategies [18].

### 4.2. Left Ventricular Function

Although electrical synchrony improved, we observed no significant acute effect on global longitudinal strain (GLS) or stroke volume (67.5 mL vs. 68.4 mL vs. 68.5 mL; *p* = NS). These results align with findings by Liang et al. (2025), who showed that AV delays optimized solely for electrical resynchronization (shortest QRS duration) may yield suboptimal hemodynamic outcomes compared to delays optimized for diastolic filling [19]. Similarly, Moubarak et al. (2020) found that the acute correction of dyssynchrony does not uniformly predict immediate hemodynamic benefit, although it may indicate long-term responders [20].

### 4.3. Left Atrial Function

Left atrial strain and ejection fraction remained unchanged across pacing strategies. Hammersboen et al. showed that CRT reduces atrial dyssynchrony and improves LA function, but these changes occur over months and are linked to LV remodeling [21]. Therefore, the absence of acute improvement in LA indices in our study supports the concept that atrial adaptation is a delayed phenomenon.

### 4.4. Mechanical Synchrony and Segmental Strain

We observed significant improvements in segmental synchrony metrics such as Peak SL and aortic valve closure time (AVC), particularly with SyncAV. These findings suggest that fusion pacing can influence contraction timing favorably even when global metrics remain stable. This observation is supported by the acute findings of Tam et al., who demonstrated that real-time ECGi-guided CRT, based on activation time reduction, correlates strongly with later echocardiographic improvements, even in non-LBBB patients [22]. However, we acknowledge that while statistically significant, the changes in Peak SL and AVC have not been validated against minimal clinically important difference (MCID) thresholds in CRT populations. Future studies incorporating anchor-based clinical metrics are needed to contextualize these findings.

### 4.5. Right Ventricular Function

Both pacing strategies improved the S’ velocity, an indicator of RV systolic function. The increase was statistically significant, suggesting possible interventricular interaction. While TAPSE remained stable, the findings support recent metanalysis that CRT can benefit RV performance, particularly in patients with preserved preload and sinus rhythm [23].

### 4.6. Methodological Considerations

This study utilized a within-subject crossover design, wherein each patient underwent all pacing modes sequentially. This design eliminates between-subject variability and increases statistical power despite a relatively small sample size (n = 27). All echocardiographic measurements were analyzed offline by two blinded, experienced echocardiographers. Measurements of strain and synchrony followed ASE guidelines using EchoPAC software (version 203) and vendor-specific algorithms, including speckle-tracking for GLS and Peak SL, and Auto LAQ for left atrial parameters. Although software-specific tools were used, consistent methodology across patients ensured internal reliability. Nonetheless, broader comparability to other platforms may require caution.

### 4.7. Comparison with Other CRT Trials

Unlike LOT-CRT strategies that involve conduction system pacing and the direct recruitment of the His–Purkinje system, SyncAV remains a fusion-enhancing extension of BiV pacing. While LOT-CRT has demonstrated superior hemodynamic outcomes in patients with wide QRS and suboptimal BiV response [24], our findings reinforce the notion that SyncAV, although less invasive, may offer an intermediate benefit—marked electrical improvement without immediate hemodynamic gains. Notably, Elliott et al. reported that BiV-endocardial pacing and LBBAP provided superior acute resynchronization and hemodynamics compared to BiV-epicardial pacing, suggesting that pacing site and substrate (scar) may play critical roles in CRT outcomes [13].

### 4.8. Clinical Implications

Our findings suggest that fusion pacing using the SyncAV^®^ algorithm provides immediate electrical benefits in CRT recipients, notably QRS narrowing and improved segmental synchrony, without corresponding acute hemodynamic changes. This aligns with prior research, including the Adaptive CRT trial by Martin et al., which demonstrated that fusion strategies can optimize electrical conduction without compromising clinical response.

Importantly, the absence of short-term improvement in left atrial (LA) function or global LV strain supports existing evidence that atrial remodeling and full hemodynamic benefit often require long-term pacing and structural adaptation.

From a clinical standpoint, our results reinforce that electrical optimization strategies like SyncAV may serve as an early step in improving CRT response. However, clinicians should not expect immediate gains in parameters such as stroke volume or GLS. Instead, these algorithms may be most useful in facilitating long-term remodeling and functional recovery—especially in patients with suboptimal biventricular pacing alone.

Furthermore, the observed improvement in RV function indices, including S′, supports recent meta-analytic findings highlighting CRT’s role in biventricular interaction and right ventricular reverse remodeling. This may be particularly relevant in heart failure patients with coexisting RV dysfunction.

In the broader context of CRT optimization, our findings emphasize the value of individualized programming. Fusion-based strategies may offer a non-invasive means to enhance electrical synchrony, especially in patients with preserved AV conduction, and should be considered as part of a multiparametric optimization approach in CRT management.

### 4.9. Limitations

This study focused on the immediate, beat-to-beat effects of different pacing modes on myocardial function. While it provides valuable insight into the acute physiological response to pacing adjustments, it does not address the long-term impact of sustained dual-chamber pacing. Potential benefits related to reverse remodeling and progressive improvement in mechanical synchrony may emerge only after extended follow-up.

This study was conducted at a single center with a relatively small sample size (n = 27), which may limit the generalizability of the findings and reduce statistical power, particularly for secondary endpoints. Additionally, while all patients had sinus rhythm and stable medical therapy, some variability in baseline left ventricular geometry and etiology may have influenced the hemodynamic response to pacing.

In particular, the presence of myocardial scar tissue in patients with ischemic cardiomyopathy could attenuate the effectiveness of resynchronization therapy. Cardiac magnetic resonance imaging (CMR), the reference standard for scar quantification, was not routinely performed in this cohort, limiting our ability to correlate scar burden with pacing response.

Although echocardiographic analyses were performed by experienced operators using standardized software and acquisition protocols, no formal inter- or intra-observer variability assessment was conducted. Finally, while our findings support the electrical benefits of SyncAV-based fusion pacing, head-to-head studies comparing this approach with LOT-CRT or conduction system pacing (CSP) strategies are warranted to determine whether early improvements in synchrony translate into durable clinical outcomes.

## 5. Conclusions

The immediate QRS narrowing observed in CRT patients—particularly with fusion pacing optimization via the SyncAV^®^ algorithm—is associated with a more homogeneous pattern of left ventricular contraction and improvements in selected synchrony parameters. However, these acute electrical enhancements do not result in immediate changes in stroke volume, global longitudinal strain, or left atrial function. Further longitudinal studies are warranted to determine whether improved electrical synchrony leads to meaningful hemodynamic benefits over time.

## Figures and Tables

**Table 1:** Baseline characteristics of the study population (n = 27).

Characteristic	Value
Age, years	71 (64–76) *
Female sex, n (%)	6 (22%)
Hypertension, n (%)	25 (93%)
Ischemic etiology, n (%)	15 (56%)
-myocardial infarction	12 (44%)
-PCI	10 (37%)
Diabetes mellitus	11 (41%)
NYHA class II/III, n (%)	15 (56%)/12 (44%)
QRS duration, ms	172 ± 17.5
Heart rate, bpm	68 ± 6
Echocardiographic parameters	
Left ventricular ejection fraction (LVEF), %	30 (22–36) *
LV end-systolic volume (LVESV), mL	149 ± 78
LV end-diastolic volume (LVEDV), mL	199 ± 82

* Values are presented as median (interquartile range) or mean ± standard deviation, as appropriate. NYHA—New York Heart Association; LVEF—left ventricular ejection fraction; LVESV—left ventricular end-systolic volume; LVEDV—left ventricular end-diastolic volume.

**Table 2:** Major findings.

Parameter	CRT OFF	BiV	Sync AV	*p*-Value	Post Hoc
QRS	160 ms (140–200)	130 ms (110–160)	120 ms (80–160)	<0.001	1 > 2 > 3
LA EF	42% (19–61)	43% (17–70)	45% (14–57)	0.725	nan
E/e′ aver	11.33 (6.67–56)	10 (6.8–46.8)	11.67 (6.5–66.2)	0.837	nan
GLS	−8.6	−8.5	−9	0.212	nan
Peak SL	109 (45–226)	90 (42–185)	94 (43–200)	0.015	1 > 2, 3
AVC	416 (279–533)	435 (359–526)	443 (360–528)	0.043	3 > 1
S′	12 (6—16)	12 (4–22)	13 (7–19)	0.026	2, 3 > 1

Values are presented as median (interquartile range) or mean ± standard deviation, as appropriate.; SyncAV^®^—Fusion pacing algorithm by Abbott; QRS—QRS complex duration on ECG; LA EF—Left atrial ejection fraction; E/e′—Ratio of early mitral inflow velocity to early diastolic mitral annular velocity; GLS—Global longitudinal strain; Peak SL—Time to peak segmental longitudinal strain; AVC—Aortic valve closure time; S′—Systolic velocity at the tricuspid annulus (tissue Doppler).

## Data Availability

The raw data supporting the conclusions of this article will be made available by the authors on request.
